# A Review of Computer-Aided Expert Systems for Breast Cancer Diagnosis

**DOI:** 10.3390/cancers13112764

**Published:** 2021-06-02

**Authors:** Xin Yu Liew, Nazia Hameed, Jeremie Clos

**Affiliations:** Jubilee Campus, University of Nottingham, Wollaton Road, Nottingham NG8 1BB, UK; nazia.hameed@nottingham.ac.uk (N.H.); jeremie.clos@nottingham.ac.uk (J.C.)

**Keywords:** machine learning, deep learning, computer aided diagnosis, breast cancer, histopathology images, classification, medical imaging

## Abstract

**Simple Summary:**

Breast cancer is one of the most commonly diagnosed diseases in females around the world. The most threatening is when cancer spreads uncontrollably to other parts of the body and can cause death. Early detection of breast cancer lowers the risk of death among patients and enables appropriate treatments to control the progression of cancer. To diagnose breast cancer, high complex visuals of the breast tissue can be collected through histopathology images that provide informative details which validate the stage of the cancer. The aim of this study is to investigate techniques applied in histopathology images in diagnosing breast cancer.

**Abstract:**

A computer-aided diagnosis (CAD) expert system is a powerful tool to efficiently assist a pathologist in achieving an early diagnosis of breast cancer. This process identifies the presence of cancer in breast tissue samples and the distinct type of cancer stages. In a standard CAD system, the main process involves image pre-processing, segmentation, feature extraction, feature selection, classification, and performance evaluation. In this review paper, we reviewed the existing state-of-the-art machine learning approaches applied at each stage involving conventional methods and deep learning methods, the comparisons within methods, and we provide technical details with advantages and disadvantages. The aims are to investigate the impact of CAD systems using histopathology images, investigate deep learning methods that outperform conventional methods, and provide a summary for future researchers to analyse and improve the existing techniques used. Lastly, we will discuss the research gaps of existing machine learning approaches for implementation and propose future direction guidelines for upcoming researchers.

## 1. Introduction

**Figure 1:**
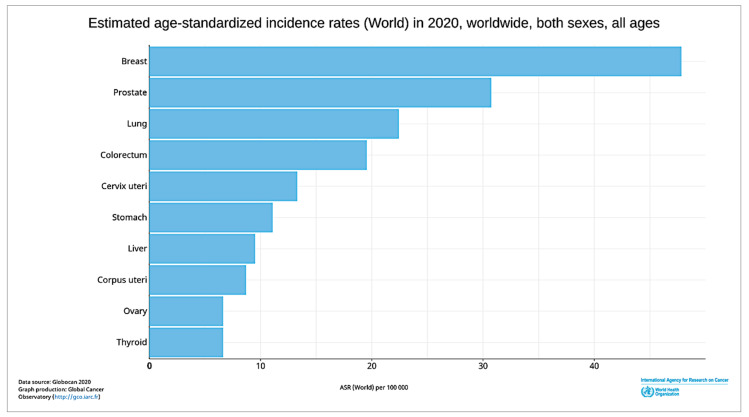
Incidence rates of cancer in 2020 [1].

The human body is formed of trillions of cells. ‘Cancer’ is a term used when a cell divides abnormally or uncontrollably, which can happen in various parts of the body. The disease type is categorised based on which part of the body cancer occurs. This situation, if left unchecked, will lead to death. Amongst the distinct types of cancer, the most common type of cancer happening in females is breast cancer. According to the World Health Organisation (WHO), breast cancer is the most frequent cancer among women, affecting 2.1 million women each year. About 627,000 women died from breast cancer in 2018, which accounted for around 15% of all cancer deaths among women [1]. In the United Kingdom, there are around 55,200 newly diagnosed breast cancer cases every year, which is about 150 every day from 2015 to 2017 [2]. From Figure 1, we can observe that breast cancer has the highest number of diagnosis incidence among all the common cancers [1].

However, it has been proven that an early detection of breast cancer can significantly increase the chances of successful treatment plan and ensure a long-term survival of the patients [3]. Statistically, if the disease is detected and diagnosed at an early stage, nearly all (98%) patients will survive for five years or more, compared to around 1 in 4 (26%) people when the disease is diagnosed at a later stage [2]. According to the most common procedure, a ‘two-week wait’ is the procedure to diagnose breast cancer [2]. The standard procedure to diagnose breast cancer by pathologists usually requires extensive microscopic assessment. Therefore, having an automated solution like a computer-aided diagnosis (CAD) system not only contributes to an easier diagnostic process, but also reduces the subjectivity in diagnosis.

**Figure 2:**
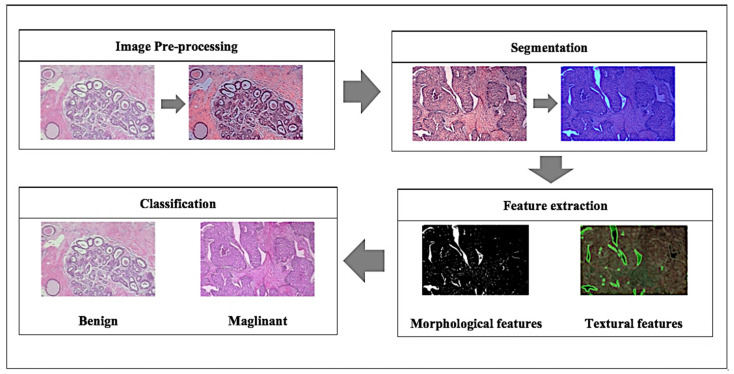
Core steps involved in a breast cancer computer-aided diagnosis system.

With the advanced development of artificial intelligence, many machine learning techniques have been applied for CAD systems. This technique can potentially outperform humans and learn more efficiently with time, therefore integrating machine learning in diagnosis can supply useful knowledge to assist pathologists in evaluating and analysing enormous amounts of medical data [4]. It could also speed up the process due to the capability to process large data much faster than manual diagnosis by a pathologist [4]. Breast cancer diagnosis can be considered as a classification problem in machine learning, in which the result indicates which class of cancer it belongs to. Fundamentally, the main steps involved in developing the core of a computer-aided diagnosis (CAD) system for breast cancer are presented in Figure 2.

Conventionally, several popular machine learning algorithms applied to classification problems include naïve Bayes [5], artificial neural network [6], support vector machine (SVM) [7], and many more. However, these algorithms might not have the ability to consider issues such as imbalanced costs of misclassification within various classes, leading to undesired consequences. Recently, deep learning methods were introduced to improve on conventional machine learning methods by extracting information automatically as part of the learning process, leading to undoubtedly better solutions [8]. Deep learning was shown to outperform state-of-the-art methods in many fields of medical imaging analysis tasks. Therefore, in this paper we will discuss and compare both approaches applied to develop a CAD system for the breast cancer.

**Figure 3:**
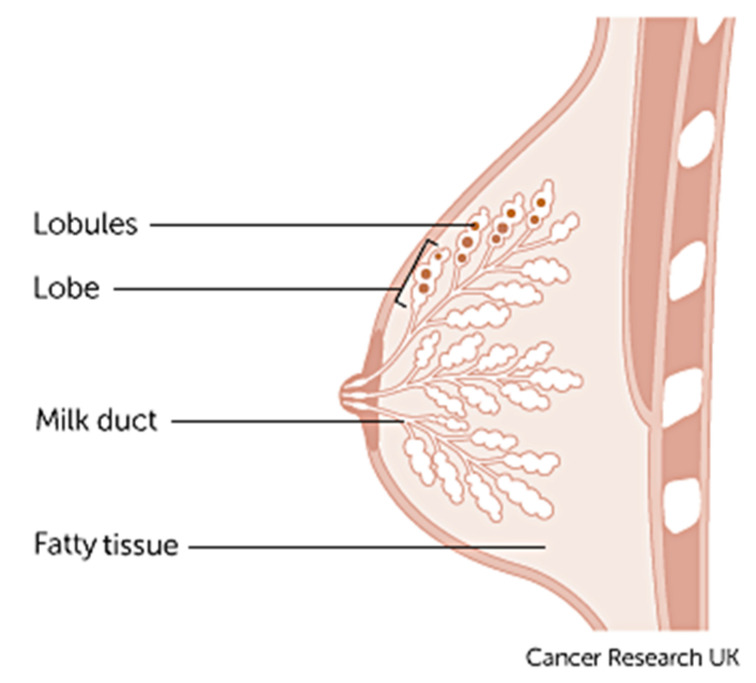
Anatomy of the breast credits to Cancer Research UK [9].

Breast cancer varies based on which part of the breast tissue becomes cancerous. Commonly, breast cancer starts in the cells that line the ducts of the breast; however, it may also grow in different areas of the breast such as the lobules, milk ducts or sometimes in between tissues, as illustrated in Figure 3 [9].

**Figure 4:**
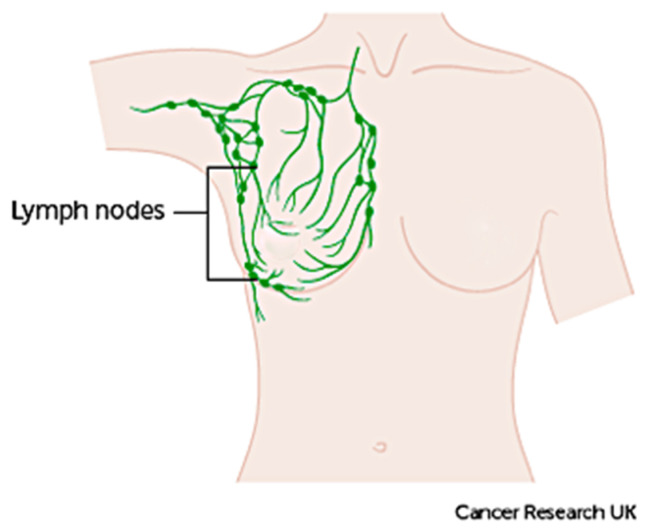
Network of lymph nodes around the breast based on a graphic created by Cancer Research UK [9].

The term ‘breast cancer’ refers to a malignant tumour that has developed from cells in the breast that are considered cancerous and cause danger to health. The stage of this cancer is usually expressed as a number on a scale of 0 through IV, with stage 0 describing non-invasive cancers that are still within their original location and stage IV describing invasive cancers that have spread outside the breast [10]. In cases where cancer is detected, but no cancer cells are visible in the lymph glands, the breast cancer is of a lower risk. When spreading occurs, it carries a substantial risk of death, meaning that the cancer cells from the breast tissue have broken away, which can be carried to nearby lymph nodes by the lymph fluid (fluid that gathers waste products and drains into veins to be removed) [10]. Figure 4 demonstrates the lymph nodes around the breast [9].

Breast cancer can be distinguished as benign (non-cancerous) and malignant (cancerous/metastatic) tumours. Benign tissue refers to changes in normal tissue of breast parenchyma, which does not relate to the development of malignancy [11]. Contrarily, malignant tissue can be categorised into two types: in-situ carcinoma and invasive carcinoma. Additionally, in some cases benign breast tumours can be further divided into four subclass types, adenosis, fibroadenoma, phyllodes tumour, and tubular adenoma, whereas malignant breast tumours can be further divided into ductal carcinoma, lobular carcinoma, medullary carcinoma, mucinous carcinoma, tubular carcinoma, and papillary carcinoma [12].

**Figure 5:**
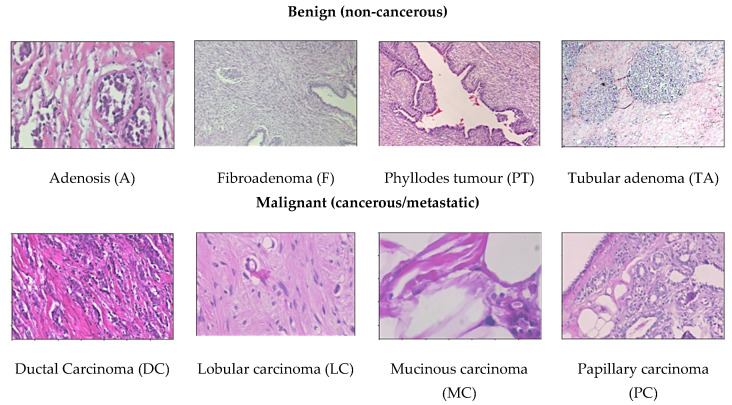
Sample of distinct types of breast cancer histopathology images from the BreaKHis dataset [15].

Histopathology (histology) image samples of breast lesions are obtained through either needles or surgical operation, which are then later processed and allocated to a glass slide to undergo a staining process. Haematoxylin and eosin (H&E) and immunohistochemistry (IHC) are the most used histopathology staining protocols [4]. This development of scanners have digitalized histopathological tissue sections and turned digital pathology into a routine practice [13]. Currently, histopathological images play a vital role in cancer diagnosis because of the large amount of information they provide for medical image analysis [14]. Whole-slide images (WSI) can have multiple regions of breast lesion tissue, whereas microscopy images are patches derived from WSI, each representing one type of breast lesion only. In this paper we have chosen to study histopathology images of breast cancer in developing a machine learning based CAD system. Figure 5 demonstrates eight classes of breast cancer from the BreaKHis dataset [15].

The main contribution of this paper is to discuss the process, methods, comparisons, and remarks on developing a CAD expert system for breast cancer. The rest of the research paper is organized as follows: Section 2 explains the publicly available datasets for breast cancer histopathology images. The process of using a computer-aided expert system using histopathology images is presented in Section 3, which includes techniques employed in (1) image pre-processing, (2a) conventional CAD methods that employ segmentation, feature extraction, feature selection (dimension reduction) and classification; (2b) deep-learning-based CAD and (3) Performance evaluation. Finally, Section 4 discusses the conclusion and future directions for researchers are given Section 5.

## 2. Datasets for Breast Cancer Classification

In the field of medical image analysis, machine learning methodologies applied for histopathological images are developing rapidly. However, there is still a demand for an automatic system to get efficient and highly accurate results [14]. To obtain a large and representative annotated dataset to develop a machine learning method for CAD system is a challenging task [16]. Recently, there has been a rise in public challenges for breast cancer diagnosis which has attracted many researchers to this area of study. This section describes various publicly accessible datasets to assist future research and development.

1. BreaKHis dataset [15]: This dataset provides 4 different magnification levels of 40×, 100×, 200×, and 400× histology images of size 752 × 582 pixels. It consists of a total number of 7909 images acquired from a clinical study from January 2014 to December 2014 in P&D Laboratory, Brazil by 82 patients. For binary classification, there are two categories of benign and malignant to determine cancer or non-cancerous. There are 1995 images (652 benign and 1370 malignant) in 40× magnification level; 2081 images (644 benign and 1437 malignant) in 100× magnification level; 2013 images (623 benign and 1390 malignant) in 200× magnification level; and 1820 images (588 benign and 1232 malignant) in 400× magnification level in the dataset. To further perform multiclassification, the dataset contains four distinct types for each breast tumours. The category benign type of breast tumour consists of adenosis (A), fibroadenoma (F), phyllodes tumour (PT), and tubular adenoma (TA). The malignant type of breast tumour consists of ductal carcinoma (DC), lobular carcinoma (LC), mucinous carcinoma (MC), and papillary carcinoma (PC). This dataset is the most used dataset by many researchers for CAD breast cancer in histopathology images [11,17,18,19,20,21,22,23,24,25,26,27,28,29,30]. This dataset can be obtained from https://web.inf.ufpr.br/vri/databases/breast-cancer-histopathological-database-breakhis/ (accessed on 16 March 2021).
2. Bioimaging Challenge 2015 dataset [31]: This dataset contains 269 images of haematoxylin and eosin (H&E)-stained breast cancer histology images with image size of 2048 × 1536 pixels. Images are provided in 200× magnification level. For binary classification, there are two categories to determine cancer or non-cancerous. To further classify, the non-cancerous categories can be categorized as normal and benign, while the cancerous ones can be categorized as in situ carcinoma and invasive carcinoma. The training set has a total of 249 images form by 55 normal class, 69 benign class, 63 in situ carcinoma class, and 62 invasive carcinoma class, while the test set has a total of 20 images with 5 images for each class. Additionally, there is an extended test set with more diversity provided with a total of 16 images available. In this extended test set, there are 4 images for each class. This dataset can be obtained from https://rdm.inesctec.pt/dataset/nis-2017-003 (accessed on 16 March 2021).
3. BACH (BreAst Cancer Histology) dataset [32]: The ICIAR 2018 challenge resulted in the BreAst Cancer Histology (BACH) image dataset, which is an extended version of the Bioimaging 2015 breast histology classification challenge dataset with similar image sizes and magnification levels [31]. The dataset has a total number of 400 images, respectively classified to a total number of 100 normal class, 100 benign class, 100 in situ carcinoma class, and 100 invasive carcinoma class. The test set has a total of 100 images without any labels. The dataset can be obtained from https://iciar2018-challenge.grand-challenge.org/ (accessed on 16 March 2021).
4. CAMELYON dataset [33]: The Cancer Metastases in Lymph Nodes Challenge breast cancer metastasis detection dataset combines two datasets collected from CAMELYON16 and CAMELYON17 challenges, with each image approximately 1 × 105 by 2 × 105 pixels at the highest resolution. The first dataset CAMELYON16 consists of a total 400 whole-slide images (WSIs) of haematoxylin and eosin (H&E)-stained lymph node sections collected from Radboud University Medical Center (Nijmegen, The Netherlands) and the University Medical Center Utrecht (Utrecht, The Netherlands). Each image is annotated with a binary label for classification, showing normal and presence of metastases tissue. There are two sets of training datasets, the first has a total number of 170 images, formed of 100 normal class and 70 metastases class, while the second has a total number of 100 images formed of 60 normal class and 40 metastases class. The test set holds a total number of 130 images. The CAMELYON17 dataset consists of a total of 1399 histology breast images. This version is extended from the CAMELYON16 which include patients testing for breast cancer from the CAMELYON16 challenge with an additional three medical centres from the Netherlands, specifically: slides from 130 lymph node resections from Radboud University Medical Center in Nijmegen (RUMC), 144 from Canisius-Wilhelmina Hospital in Nijmegen (CWZ), 129 from University Medical Center Utrecht (UMCU), 168 from Rijnstate Hospital in Arnhem (RST), and 140 from the Laboratory of Pathology East-Netherlands in Hengelo (LPON) [34]. The dataset can be obtained from https://camelyon17.grand-challenge.org (accessed on 16 March 2021).
5. PatchCamelyon (PCam) dataset [35]: Whole slide images (WSI) are computationally expensive and only require the small regions of interest (ROIs) from the entire image, therefore it would need to estimate a significantly substantial number of parameters. Thus, this version of dataset is derived from the CAMELYON dataset with a total number of 327.680 histopathologic scans of lymph node sections images, each in the size of 96 × 96 px pixels. Like the CAMELYON dataset, each image is annotated with binary label for classification, showing normal and presence of metastases tissue. The main difference and advantage of this dataset is that it is bigger than CIFAR10, smaller than ImageNet, additionally it is trainable on a single GPU to able to achieve competitive scores in the CAMELYON16 tasks of cancer detection and WSI diagnosis. PCam contributed by supplying the segmented tissue parts that separated tissue and background from the whole slide images. The dataset can be obtained from https://github.com/basveeling/pcam (accessed on 16 March 2021).
6. MITOS-12 dataset [36]: The conference ICPR 2012 supplied the MITOS dataset benchmark that consists of 50 histopathology images of haematoxylin and eosin (H&E)-stained slides of breast cancer images from 5 different breast biopsies at 40× magnification level. However, this dataset is too small to produce an exceptionally reliable performance and the robustness of the diagnosis system is limited. Therefore, an extended version of the dataset (MITOS-ATYPIA-14) was presented at ICPR 2014.
7. MITOS-ATYPIA-14 dataset [37]: The grand challenge dataset was presented at the ICPR 2014 conference, extended from the MITOS-12 challenge that provides haematoxylin and eosin (H&E)-stained slides of breast cancer images with the size of 1539 × 1376 pixels at 20× and 40× magnification level. There is a training set with a total number of 1200 images acquired from 16 different biopsies and testing set with a total number of 496 images acquired from 5 different breast biopsies. The dataset consists of a significantly diverse variation of stained images in many conditions to elevate the challenge to achieve a more exceptional performance. The dataset can be obtained from https://mitos-atypia-14.grand-challenge.org/ (accessed on 16 March 2021).
8. TUPAC16 dataset [38]: The dataset consists of a total number of 73 breast cancer histopathology images at 40× magnification level from three pathology centres in the Netherlands. The dataset is composed of 23 test images with a size of 2000 × 2000 pixels and 50 training images with a size of 5657 × 5657 pixels collected from two separate pathology centres. The images contained in the training dataset are later cropped randomly to the size of 2000 × 2000 pixels. The dataset can be obtained from http://tupac.tue-image.nl/node/3 (accessed on 16 March 2021).
9. UCSB bio segmentation benchmark (UCSB-BB) [39]: This dataset contains 50 haematoxylin and eosin (H&E)-stained histopathology images used in breast cancer cell detection with the size of 896 × 768 pixels and a ground truth table. Each image is annotated with binary label for classification, it contains half malignant class and half benign class. The dataset can be obtained from https://bioimage.ucsb.edu/research/bio-segmentation (accessed on 16 March 2021).

## 3. Computer-Aided Diagnosis Expert Systems

CAD systems have not only produced faster diagnosis results but have also emerged as an additional opinion to assist pathologists to avoid overlooking abnormal features. This automated solution can be explained in two sub-categories:

1. Computer-aided detection (CADe) systems, which detect cancer or metastatic tissue.
2. Computer-aided diagnosis (CADx) systems, which determine the distinct types of breast cancer.

**Figure 6:**
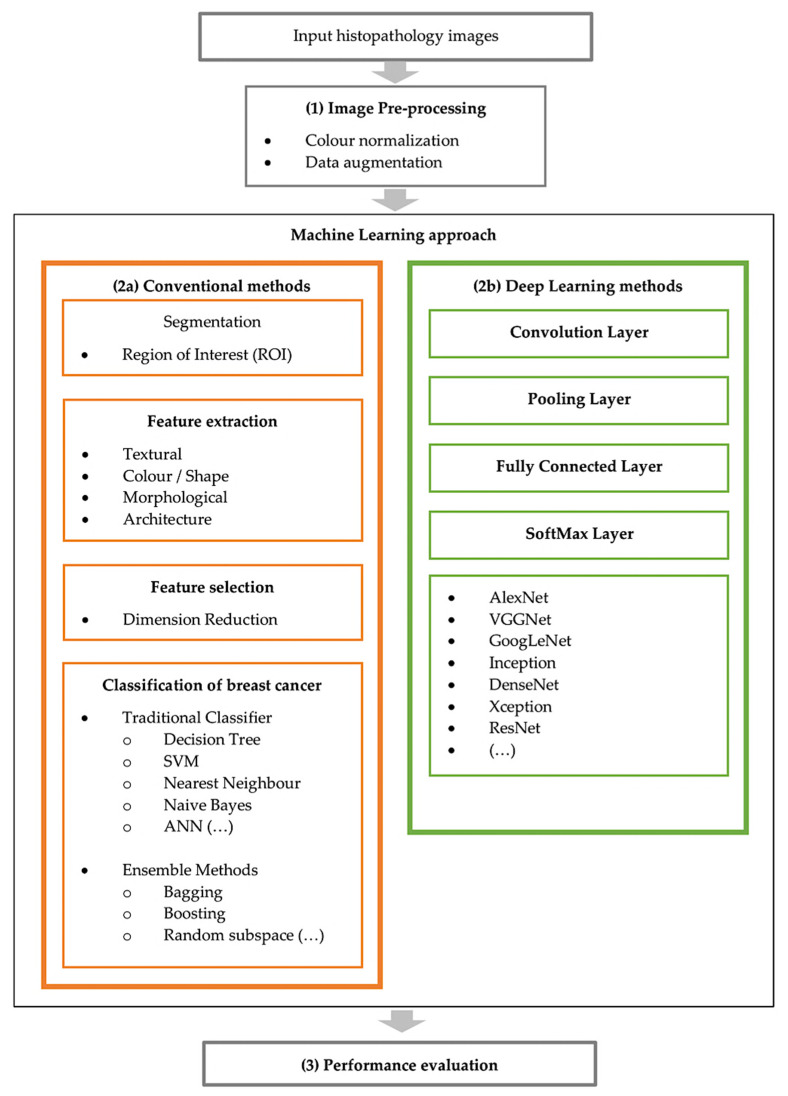
Overview structure of methods used to classify breast cancer in CAD systems.

There are two approaches in developing a CAD system which are the conventional method and deep learning method. The main difference between these two types of methods is that conventional CAD methods are a traditional approach of extracting the features from an image based on human-defined descriptors to perform classification. Deep learning CAD methods are types of automated learning that can discover representations of data automatically by transforming the input information into multiple layers of abstractions [8]. Figure 6 illustrates these two methods for CAD systems.

### 3.1. Image Pre-Processing

Image pre-processing is an effective route to apply as data preparation at the first step to make raw data more suitable for further analysis. In the case of histopathology images, the most used pre-processing technique is colour normalisation because of the colour variation obtained in these types of images and the powerful impact on the machine learning model. Data augmentation is another commonly used technique for a small dataset. In this section, the techniques of (1) colour normalisation and (2) data augmentation are presented.

(1) Colour normalisation: The inconsistent various appearances of stained sections is amongst the foremost challenges to analyse histopathological images [40]. This is because the samples are collected under various inconsistent conditions of tissue slices, preparation or image acquisition, noise arising, lightning conditions, and protocols of staining while capturing the digital image [40]. Therefore, these variations could produce samples with different colour intensities [41]. Research studies [18,42] have shown the significant effect of stain normalisation that enhances the performance of breast cancer classification. Here, a few colour normalisation techniques will be investigated by categorizing them into three types of method which are global colour normalisation, the supervised method, and the unsupervised method for stain separation.
  - Global colour normalisation: This method is suitable for histology images due to comprehensible values of autocorrelation coefficient or spatial dependency of pixel (intensity). This method separates colour and intensity information using principal component analysis (PCA) [43]. Reinhard et al.’s method was one of the first techniques, which uses a simple statistical analysis to achieve colour correction by comparing one image’s colour boundaries and choosing it as an appropriate source image as a benchmark, applying it as characteristic to all the other images [43]. It uses an unsupervised method to heuristically estimate the absorbance coefficients for the stains for every image and the staining concentrations for every pixel to recompose the images [43].
  - Supervised method for stain separation: In this method, images are converted to optical density (OD) space due to Beer’s law [44] that suggests colour stains act linearly in OD space, given in Equation (1).

(1) $$V=log\left(\frac{I_{0}}{I}\right)$$

where V represents the intensity in OD space, I represents the intensity in RGB space, and $I_{0}$ represents the illuminating intensity incident on the sample [45]. Khan et al. proposed a method to use stain colour descriptors to compute image-specific stain matrices for stain normalisation [46]. Then, stain separation is applied to obtain different stain concentration values from the image and provide a nonlinear (spline based) mapping function; meanwhile all images will be replaced using the normalised stain channels [46].

- Unsupervised method for stain separation: Training is not required because it is expected to learn itself [47]. Macenko et al. first proposed a method to use singular value decomposition method (SVD) to obtain optical density of images to perform quantitative analysis-based colour normalisation [48]. Kothari et al. then proposed a method based on histogram specification using the quantile normalisation based on distinct colour channels obtain from images to match each image to the target image histogram colour channels [49]. Bejnordi et al. later proposed an improved version which relies solely on colour features; their algorithm makes use of spatial information to achieve robustness against severe colour and intensity variations [50]. The comparison of colour normalisation methods is provided in Table 1.
  **Table 1:** Comparison of colour normalisation methods.

Ref	Proposed Approach	Method	Advantages	Disadvantages
[43]	Colour transfer algorithm	Convert the colour space of an image from RGB to lαβ [51]. Transform the background colour of images based on the target colour space. Convert images back to RGB colour space.	All images will have the same consistent range of contrast. Structure of images remains.	Stains in images are not separated properly due to the type of colour space conversion (lαβ).
[48]	Fringe search algorithm	Convert the colour space of an image from RGB to lαβ [51]. Create plane based on calculated two largest SVD. Estimate data onto that plane. Search for corresponding DOF angles. Robust predictions of minimum and maximum are calculated by the αth and (100−α)th percentile. Convert these obtain DOF angles values back to OD space.	Negative coefficient is not found in colour appearance matrix. Absence of ambiguity.	Not ideal for automated tumour detection algorithm because the DOF angle values are estimated observationally. Original images are not preserved.
[49]	Automated colour segmentation algorithm	Apply pre segmentation by extracting the unique colours in the image to obtain colour map. Include knowledges from pre segmented reference images to normalise. Apply voting scheme to evaluate on preliminary segmentation labels. Apply segmentation to new images with the multiple reference images and combine labels from previous step.	High accuracy. Robustness. Makes use of expert domain knowledge. Retains the morphology of images.	Colour map histogram distortion due to chromatic aberration. Restricted to segmentation problems with four stain colours.
[46]	Nonlinear mapping approach	Map both target image and source images to a representation, where each channel relates to a separate chemical stain. Calculate the statistics of each corresponding channel by learning a supervised classification method (RVM). Apply a nonlinear correction (mapping) to normalise each separate channel based on previous calculation. Reconstruct the normalised source image using the normalised stain channels.	Satisfactory performance overall for separating stains. Performs at pixel level to achieve superior performance.	High computation complexity. Using nonlinear correction (mapping) functions might destroy the original image structure i.e., colour histogram. Impossible to convert back to original form of an image after mapping.
[50]	Whole-slide image colour standardiser (WSICS) algorithm	Apply hue-saturation-density (HSD) colour transformation [52] to obtain two chromatic components and a density component Gather distribution of the transformation of haematoxylin and eosin (H&E). Calculate weight contribution of stain in every pixel. Convert HSD back to RGB.	Robustness. Remain spatial information of images. It is an unsupervised method capable of detecting all stain components correctly.	Losing the original background colour during the process. High processing time. Not all information of images is preserved.

Recently, due to the significant performance and stability portrayed by these proposed methodologies for colour normalisation, many researchers have adapted these popular proposed methods above as part of their colour normalisation processes. Table 2 demonstrates the methodology used by several recent research studies for breast cancer CAD systems.

(2) Data augmentation: A data-space solution to the problem of limited data by enhancing the size of training datasets to generate a better learning model [56]. Tellex et al. showed that to obtain a particularly reliable performance of CAD system on histopathology images, colour normalisation should be used along with data augmentation [57]. This procedure will imply data wrapping and oversampling over the dataset to increase the sample size of the training dataset as a limited dataset and overfitting is a common challenge [56]. These processed include various image transformations to modify the image morphology [57,58]. If we were to look at one image from a single perspective and make a determination, it is more likely to be prone to error compared to if we were to look at it from several perspectives to make the final determination. Taking this into breast cancer analysis, checking the image with several more perspectives provides a more confident and accurate answer to which class it belongs to. Thus, this procedure provides a broader interpretation to the original image. The comparison of data augmentation techniques applied by several research studies is provided in Table 3.

### 3.2. Conventional CAD Methods

#### 3.2.1. Segmentation

The segmentation process takes part to locate the edges and boundaries of regions in a histopathology image to extract the cells in the images. It can be crucial to identify the region of interest (ROI) and highlight these significant regions in the images. This procedure involves partitioning the image Ι into non-overlapping regions [61,62], as seen in Equation (2).

(2) $$\cup^{\;}Ii=I\;\;\;\;\;\;and\;\;\;\;\;\;Ii\cap^{\;}Ij=\emptyset ,\;\;\;\;\;\;where\;I\neq j$$

**Figure 7:**
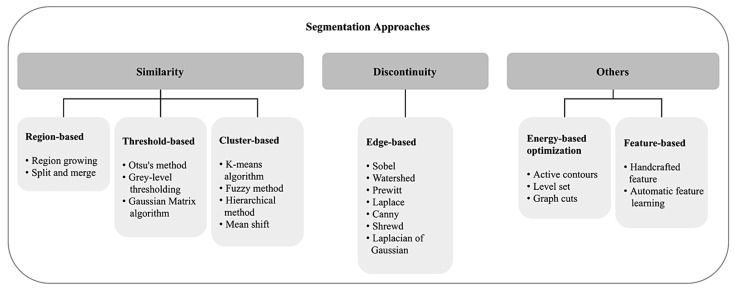
General view of segmentation approaches and techniques.

Table 4 provides a summary for each commonly used segmentation technique along with definition, advantages, and limitations. Figure 7 illustrates a general overview of the approached techniques with some examples.

**Figure 8:**
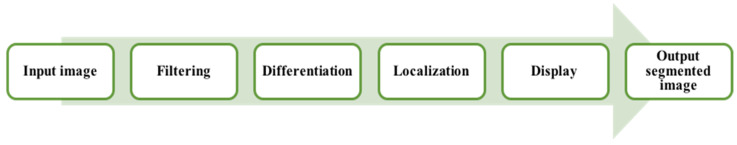
Overall process of edge-based segmentation.

- Region-based segmentation: There are two main techniques which are (1) region growing and (2) region splitting and merging. Rouhi et al. proposed the application of an automated region growing for segmentation on breast tumour histology images by using an artificial neural network (ANN) to obtain a threshold [62]. Rundo et al. used split and merging algorithms based on the seed selection by an adaptive region growing procedure [63]. Lu et al. applied a multi-scale Laplacian of Gaussian (LoG) [64] to detect the seed points and feed the filtered image to a mean-shift algorithm for segmentation, followed by some morphological operations [65].
- Edge-based segmentation: To obtain critical properties, this structural technique can be implemented in several methods for recognising the edges, such as Sobel [66], Watershed [67], Prewitt [68], Laplace [69], Canny [70], and LoG [64]. This process is illustrated in Figure 8.

George et al. applied the watershed method to extract shape and texture features of nuclei, where both features contribute to the training of an accurate nuclei classifier for breast cancer [71]. Faridi et al. used the Distance Regularized Level Set Evolution (DRLSE) algorithm for segmentation; the process includes morphological operations to detect centre of nuclei and Difference of Gaussian (DoG) filtering [72] was applied to extract nuclear boundaries [73].

- Threshold-based segmentation: To produce a less complex image, the main concept is to transform every pixel based on a threshold value; any pixels with intensity less than a threshold value/limit esteem T (constant value) will be replaced with black pixels (0), otherwise replaced with white pixels (1). The input image *g (x, y)* transformed to a threshold image *f (x, y)* can be represented mathematically as shown in Equation (3).

(3) $$f\left(x,\;y\right)=\left\{\frac{1,\;\;\;if\;g\left(x,y\right)>T}{0,\;\;\;if\;g\left(x,\;y\right)\leq T}\right\},\mathrm{where}\;T\;\mathrm{is}\;a\;\mathrm{threshold}\;\mathrm{value}$$

A few popular techniques applied in this approach are Otsu thresholding [74], grey-level thresholding [72], and gaussian matrix thresholding [72]. Zarella et al. proposed a scheme to segment breast nuclei from other parts of the cell using Otsu thresholding [75]. Saha et al. proposed an automatic nucleus segmentation on the image using histogram-based thresholding with a result of 97% accuracy in nucleus detection [76]. Moncayo et al. used Maximally Stable Extreme Regions (MSER) to perform segmentation on nuclei regions on the image’s haematoxylin contribution map, in which several thresholds are applied to the image and areas that change minimally are identified as MSER, followed by some further morphological operations [77]. A novel approach was proposed by Khairuzzaman and Chaudhury to apply a multilevel thresholding based on Grey Wolf Optimizer (GWO) using Kapur’s entropy and Otsu’s between class variances functions [78]. Sirinukunwattana et al. proposed a thresholding method to group intensity features represented by a sparse coding to create a dictionary [79].

- Cluster-based segmentation: This can be described in two clustering methods, hierarchal and partitioning [80]. Hierarchal clustering performs recursively to explore nested clusters in agglomerative (bottom to up) or divisive (top to down) ways [80], whereas partitioning clustering iteratively divides into hard clustering and fuzzy clustering [81]. Kowal et al. applied a cluster approach algorithm for nuclei segmentation from biopsy microscopic images, and achieved a high classification accuracy [82]. Kumar et al. used a k-means clustering based segmentation algorithm and mentioned that this method performs better in comparison to other commonly used segmentation methods [83]. A two-step k-means was applied for segmentation by Shi et al. to consider local correlation of pixels; they first generate a poorly segmented cytoplasm, then in a second step the segmentation does not take into account the nuclei identified during the first clustering; finally, a watershed transform was applied to complete the segmentation [84]. Maqlin et al. suggested a segmentation method based on k-means clustering algorithm to recover the missing edge boundaries based on a convex grouping algorithm, which was suitable for open vesicular and patchy types of nuclei that are commonly obtained in high-risk breast cancers [85].
- Energy-based optimization: This technique defines a cost function, and the process will minimize/maximize the function based on the object of interest (ROI) in the images. A study by Belsare et al. used a spatio-colour-texture graph cut segmentation algorithm to perform segmentation as epithelial lining surrounding the lumen [86]. Wan et al. used a combination of boundary and region information to perform a hybrid active contour method to achieve an automated segmentation of the nuclear region [87], where the energy function was defined as Equation (4).

(4) $$\varepsilon \left(\phi \right)=-\alpha \;\overset{}{\underset{w}{\int }}(Z-\mu )H\left(\phi \right)d\omega +\beta \;\;\overset{}{\underset{w}{\int }}G\left|\nabla \;H\left(\phi \right)\right|d\omega$$

where Z is the image to be segmented, H(ϕ) denotes the Heaviside function, *ω* represents the image domain, G=G(|∇ Z|) is the gradient of the image, and *α* and *β* are pre-defined weights for the balancing of the two terms. Zhang et al. proposed a three-phase level set method to set contour segments into groups and achieved high accuracy [88]. Jia et al. used a rough segmentation method to combine watershed and improved Gradient Vector Flow (GVF) Snake model to separate nuclei/cells in an image from the background to enhance the segmentation accuracy [89].

- Feature-based segmentation: Automatic segmentation based on feature learning has been commonly used for analysing medical images [90]. Song et al. used a multi-scale convolutional network to accurately apply segmentation of cervical cytoplasm and nuclei [91]. Xu and Huang applied a distributed deep neural network architecture to detect cells [92]. Rouhi et al. also proposed a cellular neural network (CNN) to perform segmentation by using genetic algorithm (GA) to determine the parameters [62]. Graham et al. proposed a deep learning method called the HoVer-Net which is a network that targets simultaneous segmentation and classification of nuclei based on the horizontal and vertical distance maps to separate clustered nuclei [93]. Zarella et al. trained an SVM model to learn the features to distinguish between stained pixels and unstained pixels using HSV colour space to identify regions of interest [94]. A summary of different segmentation approaches by several researchers is provided in Table 5.

#### 3.2.2. Feature Extraction

**Figure 9:**
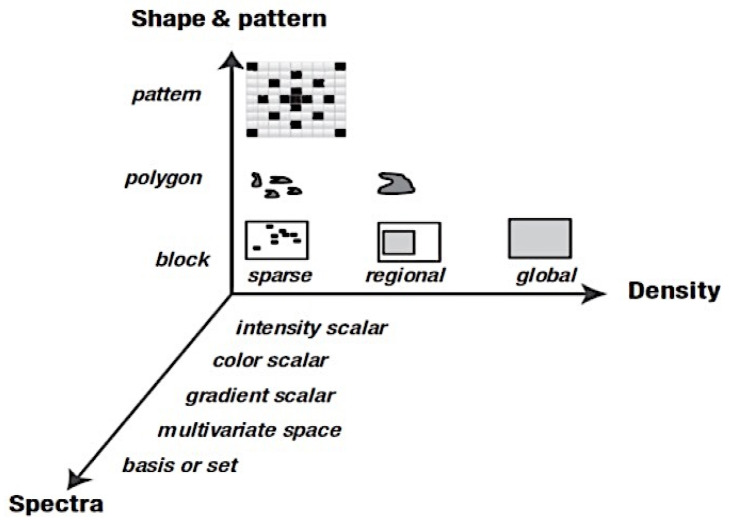
Taxonomy for feature descriptor dimensions created by Krig, S. [96].

Feature extraction is one of the essential steps to pick out a set of features that contain the most effective, relevant, and discriminating information and characteristics of ROI/entire images to be employed for classification. Overall, we can divide the image feature descriptors into three dimensions (shape, pattern and spectra, and density). From Figure 9, we can observe a feature taxonomy based on feature descriptor dimensions from the 3D axis [96].

In this section, feature extraction methods observed in the existing literature will be presented. Then, we will discuss the type of features extracted from images.

**Figure 10:**
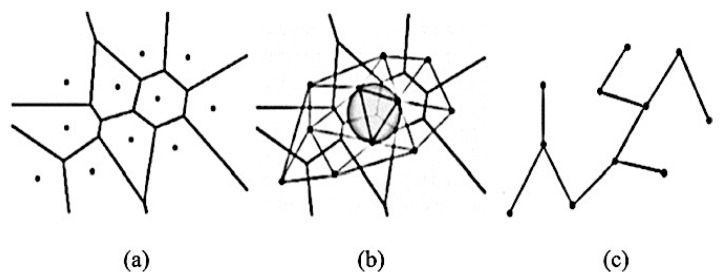
Types of graph-based topological features illustrated as (**a**) Voronoi diagram, (**b**) Delaunay graph triangulation, (**c**) minimum spanning tree [102].

- Morphological Features: Describes the details of the image regarding information in geometric aspects such as the size (radii, perimeter, and area) and shape (smoothness, compactness, symmetry, roundness, and concavity) of a cell [97].
- Textural Features: Collects information of various intensity of every pixel value from histology images by applying several methods to obtain a number of properties such as smoothness, coarseness, and regularity [97].
- Graph-Based Topological Features (architectural features): Describes the structure and spatial arrangement of nuclei in a tumour tissue [97]. When dealing with histopathological images, the arrangement and shape of nuclei is connected to the cancer development, therefore this architecture may be calculated using graph-based techniques [98,99]. There are many different topology-based features including the count of number of nodes, edges, edge length, and roundness factor to detect the tissues [100,101]. There are three types of common graph features: Voronoi diagram, Delaunay triangulation, and minimum spanning tree, as shown in Figure 10.

Belsare et al. proposed to extract the textural features such as grey-level co-occurrence matrix (GLCM), graph run length matrix (GRLM) features, and Euler number; their system was able to achieve a 100% accuracy in 70 histopathological images on a dataset from Department of Pathology, Govt. Medical College and Hospital, Nagpur, India [86]. Balazsi et al. proposed an invasive ductal breast carcinoma detector that extracts patches and a set of 16,128 features derived from multiple histograms and LBP (multiple radii) using CIELAB, grey-scale and RGB colour spaces describes each patch on a dataset from McGill University Hospital Centre pathology registry [103]. Wan et al. extracted several lots of information on multi-level features set in regards of the pixel-, object-, and semantic-level features [87]. The pixel-based features are textural features, Kirsch filters, first-order features, Gabor filters, Haralick features, HoG, and LBP. The object-based features are architectural ones represented by graphs using Voronoi diagram (VD), minimum spanning tree (MST), and Delaunay triangulation (DT). Semantic-level features capture heterogeneity of cancer biology using convolutional neural networks (CNN)-derived descriptors on a dataset from China’s No. 91 Central Hospital of PLA [87].

Recently, many authors have provided a wide range of publicly available breast cancer histopathological datasets to resolve the limitations, as shown in Section 2 Datasets for Breast Cancer Classification. Spanhol et al. provided the ‘BreaKHis’ dataset and also performed some initial experiments by applying a handcrafted method to extract textural features like local binary patterns (LBP), completed LBP (CLBP), local phase quantization (LPQ), grey-level co-occurrence matrix (GLCM), threshold adjacency statistics (TAS), parameter-free threshold adjacency statistics (PFTAS), and one key point descriptor, named ORB [15]. Sudharshan et al. investigated the parameter-free threshold adjacency statistics (PFTAS) features feeding into a multiple-instance learning (MIL)-based nonparametric classifier; their results achieved the highest patient recognition rate (Prr) on the BreaKHis dataset [30]. Gupta et al. used a set of colour-textural features including Gabor filters features, wavelet features, and local binary patterns (LBP) features to be fed into an ensemble classifier; their classification results achieved a 90.32% accuracy using the BreaKHis dataset on 200× magnification [104]. Chan and Tuszynski applied fractal dimension features for breast cancer detection; their results show that these features perform well at a magnification of 40× to distinguish malignant and benign tumours on the BreaKHis dataset [23].

Kumar et al. extracted various biologically interpretable and clinically significant shapes as well as morphology-based features, which include the grey-level texture features, colour-based features, colour grey-level texture features, Law’s Texture Energy based features, Tamura’s features, and wavelet features on a dataset with a total of 2828 histology images (histologyDS2828) [83]. Rezaeilouyeh et al. mentioned that wavelet features do not have directional sensitivity, which makes them unsuitable for detecting directional features, thus they proposed to use shearlets instead [105]. The authors proposed to perform calculation of shearlet coefficients to extract textural features to obtain them as primary features before feeding it to a CNN training stage for classification on the UCSB-BB dataset [105]. On the same dataset, Anuranjeeta et al. applied shape and morphological features of cells and achieved a result of 85.7% accuracy among 70 images [106]. On the same dataset, Bruno et al. applied a curvelet transform to handle multi-scale using a local binary pattern (LBP) algorithm to extract features from curvelet coefficients [107]. Moncayo et al. proposed a set of extracted features named bag of features (BoF) from the multi-scale textural features describing the segmented nuclei region, that is then assigned to the most similar atom of a previously learned dictionary using k-means algorithm on the National Cancer Institute: The Cancer Genome Atlas dataset [77].

Based on research, the most frequent applied hybrid techniques for cancer classification are combining morphological and textural features, for instance the work by [66,108]. Gandomkar et al. applied a hybrid approach of using segmentation-based and texture-based methods to extract features to obtain features that can discriminate between the different cancer classifications on the MITOS-ATYPIA-14 dataset [109]. Lu et al. extracted a total combination of around 142 morphological and textural features, which included the size, mean, stain’s standard deviation, sum, entropy, and mean of gradient magnitude image, 3 Tamura texture features, 44 grey-level run-length matrix-based textural features, and 88 cooccurrence matrix-based Haralick texture features on the MITOS-ATYPIA-14 dataset [65]. Khan et al. proposed to extract textural features of geodesic means of region covariance (RC) descriptors by calculating RC descriptors for different segmented regions, whereas a single descriptor for the whole image is derived by the geodesic geometric mean of these calculated RC on the MITOS-12 dataset [110]. Maroof et al. proposed a method of using hybrid feature space to combine colour features with morphological and texture features, and then changed the colour channel to calculate normalised and cumulative histograms in the wavelet domain on the MITOS-ATYPIA-14 dataset [111]. On the same dataset, Wan et al. applied a dual-tree complex wavelet transform (DT-CWT) to describe the images in the context of mitosis detection in breast cancer and the generalized Gaussian distribution and symmetric alpha-stable distribution parameters were used as features [108]. Tashk et al. combined features of LBP, morphometric, and statistical features extracted from mitotic candidates on the MITOS-12 dataset [112].

Recently, Mahmood et al. proposed a new methodology to imply post-processing techniques using feature extraction of HOG, LBP, statistical, and colour features to refine the detected mitosis cell as accepted or rejected through a threshold value based on the extracted features on the MITOS-ATYPIA datasets [59]. Bardou et al. developed two approaches where the first approach was an extracting local descriptors of dense scale invariant feature transform (DSIFT) features and speeded-up robust features (SURF) to be encoded by two coding models (bag of words and locality constrained linear coding) on the BreaKHis dataset [26].

#### 3.2.3. Feature Selection (Dimension Reduction)

Feature selection is the selection of a subset of the relevant features used in the model construction [113]. In machine learning, what we want is to avoid feature redundancy and the ‘curse of dimensionality’ problem. The ‘curse of dimensionality’ suggests that the training data have an exceptionally low density and lead to inability to promise an accurate estimation result, which defeats the purpose of training for a high accuracy classification model. This phenomenon will eventually impact the generalization performance in a negative way, for example, unstable estimation, overfitting issues. and local convergence; the large estimation error can easily compromise the prediction advantage provided by their greater representation power [114].

Therefore, this process is crucial because popular classification methods such as artificial neural network (ANN) and support vector machine (SVM), which are highly efficient for classification problems, tend to be sensitive to the dimensionality of data [115]. Additionally, data that consist of complex features reflect on quality-related issues such as the presence of noise, outliers, missing or duplicate data, and data that are biased or unrepresentative [116]. This process to reduce dimensionality can significantly eliminate irrelevant features, while the reduction in noise in machine learning contexts can produce a more robust learning model due to the association of fewer features [116].

One of the most common traditional approaches for this process is constructing new dimensions by mapping the original feature space into a new feature space with reduced dimensions. Common techniques used in this process are principal component analysis (PCA) and using a Pearson correlation matrix to construct a hierarchical representation of the data [115]. Other techniques include linear discriminant analysis (LDA), independent component analysis, and manifold learning. These techniques take the auto-covariance to solve the problem by transforming the high dimensional correlated feature set to a reduced feature set with lower dimensions.

However, these traditional approaches focus on choosing the most relevant features but disregard the fundamental interdependent structure of the features [117]. Recently, popular approaches have used heuristic search methods to select essential features from original feature space by applying methods like genetic algorithm, simulated annealing, boosting, and particle swarm optimization. In [117] and [118], the authors proposed to apply a particle swarm optimizer (PSO) as the feature selection method in reducing the high dimensionality. In [119], the authors applied a genetic algorithm (GA) to select the best features and perfect parameter values of the machine learning classifiers. These recent proposed methods focus on the disregarded fundamental interdependent structure of the features from traditional features selection method. Tambasco Bruno et al. reduced their feature space by using an analysis of variance (ANOVA) [107].

#### 3.2.4. Classification

The final set of features will then be fed as input to a classifier to estimate the breast cancer classes. The following are a few commonly applied classification methods.

- Nearest Neighbour: A non-parametric approach which falls under supervised learning widely used for both pattern recognition and classification applications [120]. The algorithm predicts each new point being input to the closest distance frame arrival point in the data; the calculation for distance varies but Euclidean distance is a common approach [121]. Let *p* and *q* be two datapoints of n-dimensions, then distance between x and y can be expressed by Euclidean distance shown in Equation (5).

(5) $$D\left(p,\;q\right)=\sqrt{\overset{n}{\underset{i=1}{\sum }}{\left(q_{i}-p_{i}\right)}^{2}}$$

Then, the algorithm compares the distance between points and classifies it into different categories [121]. Kumar et al. applied a k-nearest neighbour classifier to classify cancer and non-cancerous biopsy breast images, and have suggested that this classifier performs the best among their studies [83]. Murtaza et al. experimented with six different machine learning classifiers and showed that the KNN algorithm performs the best [20].

**Figure 11:**
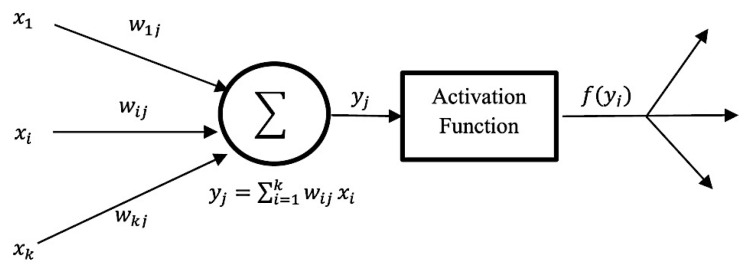
Basic structure of feed-forward ANN [127].

**Figure 12:**
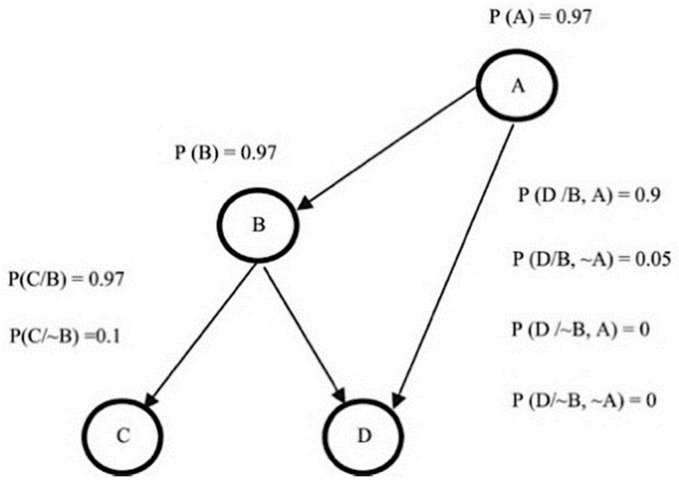
Bayesian network (BN) graph representation [130].

- Support Vector Machine (SVM): Vapnik et al. proposed this method which works by mapping input information (feature vectors) to a higher dimensional space to obtain a hyperplane that can separate the labels/classes [122]. An optimal hyperplane can be obtained by maximizing the distances between support vectors (the data points closest to the boundary of the class) of two classes [123,124,125]. Recently, several research studies on breast cancer using histopathology images were performed by applying SVM classifiers [15,30,31,54]. Korkmaz and Poyraz proposed a classification framework focusing on minimum redundancy, maximum relevance feature selection, and least square SVM (LSSVM); their results claimed to be 100% accurate with only four false negatives for benign tumours in a three-class problem; however, no further evaluation was performed [126]. Chan and Tuszynski applied SVM classifier on their fractal features; their results achieved 97.9% F-score for magnification level 40× on the BreaKHis dataset [23]. Bardou et al. have also applied an approach of SVM to classify the images using handcrafted features [26].
- Artificial Neural Network (ANN): ANN is inspired by human perception that can models complex nonlinear functions. The basic architecture of ANN starts by receiving input data $x_{i}$, calculating each of the pieces of input information by multiplying to its corresponding weight $w_{\mathrm{ij}}$, and obtaining a weighted output f (*x_j_*), with the support of a defined activation function until reaching the output layer. Figure 11 below demonstrates the basic structure of a single neuron in a feed-forward ANN [127]. Kassani et al. applied a multi-layer perceptron classifier on four different benchmark datasets and achieved the highest accuracy of 98.13% [19].
- Decision Tree: A decision tree algorithm is a supervised learning method for classification derived from the concept of ‘divide and conquer’ methodology. A complete decision tree is built based on feature space and labels; every new prediction will traverse from the root to the leaf node to produce an output. Asri et al. applied classification by using the C4.5 algorithm, an approach with a total of 11 features, and obtained 91.13% accuracy [128]. The extreme gradient boosting (XGBoost) is a new tree-based algorithm that has been increasing in popularity for data classification recently, and has proved to be a highly effective method for data classification [129]. Vo et al. have also applied gradient boosted trees as their breast cancer detection classifier [18].
- Bayesian Network: Bayesian network (BN) calculates probabilistic statistics to form a representation of relationships among a set of features space using an acrylic graph as shown in Figure 12, along with the value of conditional probabilities for each feature [130]. This type of classifier is commonly used for calculating probability estimations rather than predictions [116].
- Ensemble Classifier: This approach simply combines a few classifier methods instead of using a single classifier to produce a more accurate result. Commonly used methods to build an ensemble classifier are bagging, boosting, and random subspace method [131]. T.K. Ho proposed a random subspace classifier, in which a random feature subset is picked up from the original dataset for training each classifier; a voting scheme is then applied to produce a unique output from the from all the outputs in the combined classifiers [132]. Alkassar et al. applied an ensemble classifier that chooses the maximum score of prediction that includes a combination of decision tree, linear and quadratic discriminant, logistic regression, naive Bayes, SVM, and KNN [22].

### 3.3. Deep Learning CAD Methods

Following the recent advancements of deep learning (DL) that have shown a broad potential with state-of-the-art performance, many researchers have been approaching the process of feature extraction and selection using this automated technique. This improved approach combines learning and decision making by applying unsupervised learning upon different deep neural network architecture designs. It combines learning the features in histopathology images and classifying the images in one high complex architecture model. This process is often referred to as a black box and it can be complex to understand how deep learning works, i.e., how did the model come to this decision and what was involved in the learning process.

**Figure 13:**
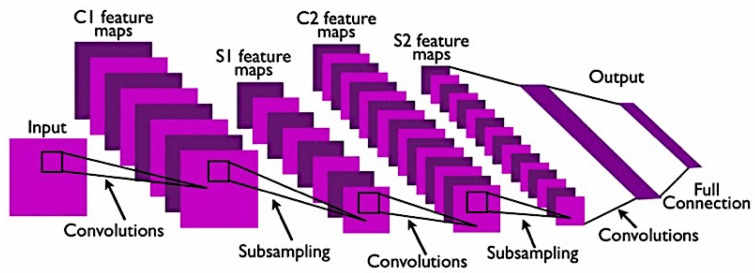
Basic structure of a CNN architecture [134].

The deep learning approach is based on convolutional neural networks (CNN) to enable a deeper level of exploration and broaden the capability of a model to perform classification on breast cancer histology images. They are able to build a complex level of non-linear mapping of input and output by utilising cascaded convolutional layers. They are considered as a unique type of neural network where instead of having weights for each input, the weights are shared and are convolved across the input as a moving window [133]. They are computational models that are composed of multiple processing layers to retrieve features from raw data with multilevel representations and hierarchical abstraction [8]. A typical CNN consists of convolutional layer, activation function, pooling layer, and output layer. An example of a standard CNN model architecture with two feature stages is shown below in Figure 13 [134].

To simplify, convolution is a signal processing operation which easily computes as a discrete spatial processing operation [121]. Recently, there have been several popular deep-learning-based models that improved the CNN model, such as AlexNet [135], VGGNet [136], GoogLeNet [137], Inception [138], DenseNet [139], Xception [140], and ResNet [141]. There are two ways to implement the method: (1) training from scratch and (2) transfer learning.

**Figure 14:**
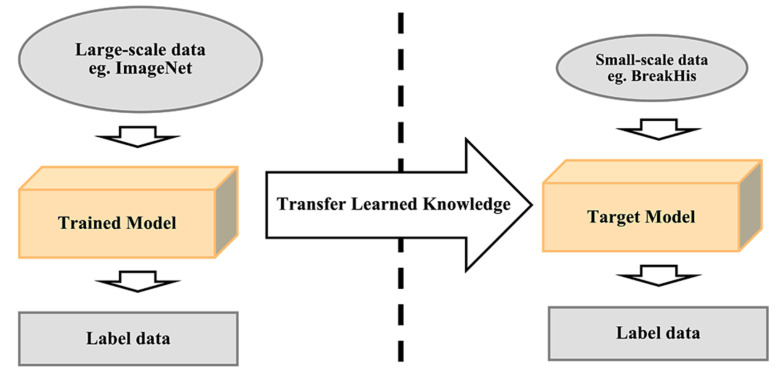
Transfer learning approach.

- Training from scratch: This method requires a large amount of input on histopathology images of breast cancer to train the CNN model. It requires more effort and skills to achieve a reliable performance CNN model when it comes to selecting hyperparameters such as learning rate, number of layers, convolutional filters and more, which can be a challenging task. This implementation also requires a high GPU processing power to perform training as CNN training can be time consuming because of the complex architecture [142].
- Transfer learning: Most publicly available datasets for breast histology images are considered as small datasets for training a deep learning model, which can be highly prone to overfitting due to the inferior performance of generalizability. The transfer learning method provides a solution to this by performing transfer knowledge tasks on the model based on a source domain that provides a large amount of sample data to the target domain. Pre-trained models can sufficiently prepare the small-scale histology dataset in a deep learning model. It can be used to: (1) perform as a baseline model, which uses the architecture of the pre-trained network and builds the model from scratch by random initialization of weights [143]; (2) perform as a feature extractor, which extracts key features and the outputs which go into the convolutional base are fed directly to the classifier without modifying any weights or convolutional parameters [143]; and (3) perform fine tuning where weights will be passed into the designed network from the pre-trained network by fine tuning the layer or performing partial training of the network [143]. Figure 14 illustrates the transfer learning approach.

Bayramoglu et al. proposed two different CNN architectures: single-task CNN is used to predict malignancy and multi-task CNN is used to predict both malignancy and image magnification level simultaneously [17]. Gandomkar et al. proposed a two-step classification in which they first used a deep residual network (ResNet) with 152 layers trained for classifying patches from the images as benign or malignant for each magnification factor [27]. Then, they used the same pre-trained model to further classify the breast cancer sub-classes. Han et al. proposed a method class structure-based deep convolutional neural network (CSDCNN) based on GoogLeNet for eight-class classification of breast histopathological slides and have shown that their accuracy was higher for fine-tuning in comparison with training from scratch [25]. Spanhol et al. also adopted AlexNet and achieved a better result than a machine learning model trained with hand-crafted textural descriptors [24]. Alom et al. proposed a binary and multi classification for breast cancer methods using the Inception Recurrent Residual Convolutional Neural Network (IRRCNN) model and achieved 99.05% (for binary) and 98.59% (for multi) classification [11].

Toğaçar et al. proposed a novel method called BreastNet using CNN model architecture that adopted a multi-layer perception (MLP) as classifier [21]. Mahmood et al. performed a score-level fusion of Resnet-50 and Densenet-201 for classification [59]. Bardou et al. experimented with a second approach to apply a CNN model, and their results showed that deep learning approaches outperformed handcrafted features [26]. Sudharshan et al. have also shown their record of achieving the highest patient recognition rate (Prr) using a multiple-instance learning-based convolutional neural network (CNN) [30]. The research by Rakhlin et al. applied ResNet-50, InceptionV3, and VGG-16 models for feature extraction and a gradient boosting tree as classifier in their proposed methodology [53]. Shallu and Mehra applied transfer learning and demonstratef that pre-trained CNNs are good substitutes for the CNNs trained from scratch for the diagnosis of breast cancer using histopathology [60]. This is because training a CNN from scratch might take a lot more time, complexity, and effort to fine tune the model, especially if it has limited numbers of samples to train, whereas a pre-trained model does not suffer from this limitation. With this, a pre-trained CNN on the ImageNet [135] database provides a larger sample to feed into a CNN model to extract features more accurately and efficiently on histopathological images [144].

Cai et al. adopted modified faster-RCNN (regional convolutional neural network) for detecting mitosis cells using the Resnet-101 network pre-trained on ImageNet database to extract features for classification [145]. Mahmood et al. has also adopted the region-based CNN technique named Faster R-CNN [146] to perform detection of mitotic cells in breast cancer histology images [59]. The general architecture of Faster R-CNN consists of an extracted feature map from input image, followed by the generation of region proposal network (RPN) [147] and a classification network of deep CNN that detects the final mitotic cells as output. Vo et al. proposed a model called Inception-ResNet-v2 that combines CNNs of Inception and ResNet to train and extract visual features from multi-scale images to achieve both global and local features from breast tumours and feed them into a gradient boosting classifier [18]. George et al. proposed an approach for breast cancer diagnosis, which extracts features from nuclei based on a pre-trained set of CNN, namely, AlexNet, ResNet-18, and ResNet-50, on random patches obtained from histology images and finally classifies them with a SVM classifier [29]. Another study by Spanhol et al. proposed a method that combines a modified AlexNet and DeCAF [148] (or deep) features extraction that is based on reusing a previously trained CNN only as feature vectors, which is then used as input for a classifier trained only for the new classification task [149]. A method named Biopsy Microscopic Image Cancer Network (BMIC_Net) by Murtaza et al. has applied pre-trained AlexNet as feature extractor [20].

Budak et al. proposed a novel method that uses a fully convolutional network (FCN) transform from AlextNet as an encoder for high-level feature extraction; the output of the FCN will then be transformed to a one-dimensional sequence for classification using Bi-LSTM [28]. A recent model named Long Short-Term Memory (LSTM) [150] based on a recurrent neural network (RNN) was introduced and has increased in popularity due to its powerful ability. The authors adopted this architecture that combines a bidirectional RNN (Bi-RNN) that handles two sources of information and LSTM for classifying breast cancer [28]. Alkassar et al. used the Xception and DeseNet to perform extraction on shallow and deep features from breast histology images [22]. Araujo et al. combined a CNN model to extract features and a SVM classifier to perform breast cancer classification [31]. One of the most promising developed deep learning models was the lymph node assistant (LYNA) algorithm based on Inception-v3 by the researchers of Naval Medical Center San Diego and Google AI [55]. They adopted the Inception-v3 network because this model has been shown to achieve greater than 78.1% accuracy on Stanford’s ImageNet dataset. Their results have successfully achieved a receiver operating characteristic area under curve (AUC) of 99% and a tumour-level sensitivity of 91% at 1 false positive per patient [55].

### 3.4. Performance Evaluation

To demonstrate the effectiveness of a CAD system for breast cancer diagnosis, it is important that we can evaluate our approaches to understand the performance of the system quantitatively as well as inspecting the underlying problems to be improved. In medical imaging diagnostic tests, sensitivity and specificity parameters are widely used to evaluate performance [40]. Other commonly used metrics for diagnosis evaluation are F1-measure [19,23,59,84,148,151], precision [19,59,60,148], accuracy [11,18,19,20,21,22,24,25,26,27,28,29,31,53,60,83,128,130,131] and receiver operating characteristics (ROC). The parameters are mostly calculated depending on the true positive (TP), true negative (TN), false positive (FP), and false negative (FN), which are the numbers of pixels corresponding to the parameters. To explain these variables, the true positive (TP) implies the number of patients who are predicted to be suffering cancer and are suffering. True negative (TN) implies the number of patients predicted to be not suffering cancer and in fact they are not suffering. False positive (FP) implies the number of patients who are predicted as cancer patients but in fact they are not suffering from cancer. False negative (FN) is the number of patients predicted as not cancer patients but in fact, they are suffering from cancer [40]. The following shows each parameter used for evaluating the performance of a classification model and its calculation formula [106].

- *Sensitivity* represents the percentage of positive numbers of samples classified correctly. The formula to calculate this is shown in Equation (6).

(6) $$Sensitivity\;\left(\%\right)=\frac{TP}{TP+FN}\times 100$$

- *Specificity* represents the percentage of negative numbers of samples classified correctly. The formula to calculate this is shown in Equation (7).

(7) $$Specificity\;\left(\%\right)=\frac{TN}{TN+FP}\times 100$$

- *Accuracy* represents the percentage of correct classification rate. The formula to calculate this is shown in Equation (8).

(8) $$Accuracy\left(\%\right)=\frac{TP+TN}{Number\;of\;Samples}\times 100$$

- *Precision* also known as PPV (Positive Predictive Value) represents the statistical variability measurement (total number of positive results). The formula to calculate this is shown in Equation (9).

(9) $$Precision=\frac{TP}{TP+FP}$$

- *Recall* represents the proportion of negative numbers of samples classified correctly. The formula to calculate this is shown in Equation (10).

(10) $$Recall=\frac{TP}{TP+FN}$$

- *F1-measure* represents the weighted mean of precision and recall. The formula to calculate this is shown in Equation (11).

(11) $$F1-measure=2\times \frac{precision\times recall}{precision+recall}$$

Besides the evaluation metrics, another useful technique in visualizing the performance of a classifier, specifically multiclassification, is by using receiver operating characteristics (ROC). This type of visual is represented in a two-dimensional graph to generate information on the trade-off of the true positive rate (sensitivity) and the false positive rate (1-specificity) within different thresholds. When evaluating a multi-class problem, each class generates different ROC graphs for comparison. To understand the ROC curve, we examine the area under the curve (AUC) to determine the capability of the features extracted for training a classifier. The larger area AUC indicates reliable performance of the model.

Recognition rate is also calculated to represent the multi-class performance on machine learning algorithms by measuring the patient-wise diagnosis [24]. The parameter is calculated as Equations (12) and (13).

(12) $$Patientscore\left(P_{s}\right)=\frac{Correctly\;classified\;cancer\;images\;of\;the\;patient}{Total\;number\;of\;cancer\;images\;of\;the\;patient}$$

(13) $$Patient\;recognition\;rate\left(Prr\right)=\frac{\sum^{\;}P_{s}}{Total\;number\;of\;patients}$$

Table 6 demonstrates the comparison of different methods, datasets used, and evaluation results by different researchers for breast cancer diagnosis systems.

## 4. Discussion and Conclusions

In this review paper, various techniques and approaches applied in every process of a CAD system have been discussed. In this section, we will discuss the importance of each process in developing a CAD system, the impact of the CAD system, and applicability of the system in the real world. To build a reliable CAD system, histopathology images that are being fed into a machine learning model perform better when they are refined and normalised. However, this process should not demolish any key features and biological tissues contained in the histopathology images. Diverse quality and intensities in images will directly affect how a machine learning model learns. Therefore, the consistency and accuracy of a machine learning CAD system depends highly on image qualities.

When it comes to segmentation, one of the critical conditions is to consider the problem of overlapping and obstructed boundaries in a histopathology image. A good segmentation technique will resolve this issue without demolishing any geometrical features. Based on our review, we can conclude that machine learning approaches perform better in tackling these issues in segmentation, for example using clustering, energy optimization, and feature-based techniques. As discussed earlier, the most common descriptive features extracted from histopathology images are morphological features, textural features, and graph topological features. Then, feature selection is applied to reduce the dimensionality of the set of features extracted to only select and focus on features that produce high impacts on the classification task. However, another approach is to use CNN topology to automatically learn the features from a histopathology image which is considered as a deep learning approach for feature extraction and selection. This approach for feature learning has been observed to perform better than manual descriptive features.

Based on this review study, the classifiers with the highest accuracy are SVM, ANN (CNN), and ensemble learning with ranges from 97.13–99% accuracy. Of course, the previous steps and design methodology of each of these approaches vary, but these classifiers are the ones that significantly contributed to the final performance. SVM models are highly efficient for non-linear decision boundaries with various kernels to select from. They are also high robustness techniques to tackle overfitting issues and dealing with high dimensional space. CNN derived from the ANN category are autonomous solutions that learn and gather information and knowledge from the images to make a decision. They are a powerful tool in analysing and processing data from grid-like topology [8] which includes images at pixel level. Ensemble learning is a robust approach as it uses multiple classifiers instead of one. It solves the issues of bias and invariance in classification task, which provides a more reliable output considering multiple factors.

The achievement and contribution of a CAD system impacts pathologists in examining breast cancer in several ways. As the process to examine a histology image requires time and effort under the microscope, it can be challenging as this type of visual contains highly complex patterns for a human eye to examine. Each process of a CAD system not only assists visualisation for pathologists, but it also provides a verification on each decision made. The impacts are discussed as follows:

- Image enhancement: Original histology images may contain visuals like noise, colour variation, intensity variation, low pixelation or more because of the staining processing during image acquisition. It is challenging to focus on the target area; therefore, image processing plays a role in standardising and improving the quality of histology images.
- Detecting the cells or nuclei: Segmentation procedure assists in locating and identifying every cell in the image. This plays a role in obtaining the accurate region of interest to further measure the existence of cancer in the cell.
- Learning the features: This process of feature extraction provides the geometrical information of the detected cell which will be later considered as knowledge to determine the possibility of cancer. The CNN approach on this matter provides a robust solution with automated learning.
- Justification on diagnosis results: There always exists a situation where pathologists might examine an incorrect result due to several factors such as lack of experience, heavy workload, human error, or miscalculation. Thus, a CAD system can provide a second perspective on or verification of the diagnosis results by pathologists under the microscope assessment.
- Fast diagnosis results: As discussed in this paper, one of the benefits of a CAD system is to help breast cancer patients in early diagnosis to treat it before it progresses to more advanced stages. Pathologists often face challenges while diagnosing breast cancer because it requires an extensive amount time, effort, and process to perform microscope examination on histology images, therefore a CAD system can efficiently provide a faster solution.
- Improve productivity: The advancement of machine learning techniques produces higher productivity in a pathologist’s microscope examination and possible reduction of the number of false negatives associated with morphologic detection of tumour cells for deep learning techniques [55].

Although CAD systems provide an optimistic aspect in assisting the medical image analysis and better performance, there exist several research gaps on using a CAD system in a real-world environment. Due to these limitations, it is challenging to adopt these tools in a real-world environment.

- Data limitations: Working with complex and large amounts of medical data can be challenging as they require high processing power and huge memory storage. Machine learning, especially deep learning, requires a large amount of data to train the model to produce a reliable and correct result. Some of the research papers acquired small datasets from private institutions, which are more likely to perform differently when being used in the real-world hospital environment. For publicly available datasets, most of them are considered as small datasets which are also most likely not applicable when it comes to performing in a real-world environment. Looking at the largest public dataset, for example the BreaKHis dataset, it does not satisfy the condition of a dataset with enough patient samples. Therefore, existing CAD systems do not have sufficient knowledge learned that is ready to be applicable in the real-world environment.
- Bias and imbalance class: This problem among datasets can lead to undesired classification for the diagnosis result. When a CAD system is built upon a dataset with imbalanced classes, the results will be more likely to be biased and therefore produce wrong diagnosis. When a trained model is biased to a specific class due to the imbalanced dataset it destroys the reliability of a CAD system because it will increase the rate of wrong classification. There are solutions to deal with problems like these by applying oversampling, undersampling, and algorithm-level methods [152]. Therefore, there are insufficient investigations performed on solutions that show significant improvement for imbalanced data to be able to practically use it in hospitals.

This paper has presented the detailed process of designing a machine learning computer-aided diagnosis expert system for breast cancer on histopathology images using both conventional and deep learning approaches. Publicly available histopathology images have also captivated the interest of many developers and researchers in exploring the possibilities in the datasets. Machine-learning-based CAD systems have contributed a promising performance when compared to a diagnosis performed by a pathologist using a microscope. The advancement of deep learning has also remarkably outperformed the conventional approach on feature learning and capability of a CAD system. Analysing medical datasets of breast histopathological images is a challenging task due to differences and artifacts during image acquisition and because of the complex images. Therefore, techniques developed for analysing breast histology images require robustness to overcome all underlying variations. This review paper has explored the most recent developments in breast cancer diagnosis systems and provides a comparison overview of accuracy, benefits, disadvantages, and techniques employed by different researchers. A general review has been presented on techniques applied for classifying breast cancer, existing challenges, and the future direction of computer-aided diagnosis (CAD) systems for breast cancer.

## 5. Future Directions

Although many research studies presented in this paper have showed particularly reliable performance, there are still further possibilities to be explored in the future scope to further improve breast cancer diagnosis systems.

- Recently, the investigation and proposed CNN models have been increasing to provide an efficient solution to solve task-specific problems. In the future, there is always space for a new and more powerful CNN model that combines and utilises all the existing CNN’s good characteristics to be discovered. For breast cancer classification, specifically a model that performs segmentation on cancer and non-cancerous regions.
- Most research studies focus on the indicators of accuracy and performance metrics while developing a diagnosis system. However, when it comes to the applicability in real-world hospital environments the performance is undefined. Problems like class imbalance and large-scale diagnosis systems require extensive investigation in unpredictable real-world environments to obtain reliable CAD systems. Therefore, further investigation needs to be performed and will require many years of clinical practice of a CAD system in the real-world environment to constantly adapt and improve to be able gain credibility for clinical adoption in the future.
- Currently, the development of pre-trained CNNs on histopathology breast cancer image datasets does not exist. Most of the current research studies apply feature extraction that uses pre-trained CNNs on the general ImageNet data. Therefore, future researchers can explore building a large-scale pre-trained CNN focusing on breast cancer histopathology images that is task specific to assist breast cancer diagnosis.
- In recent research studies, the authors in [26] have applied feature descriptors of scale invariant feature transform (DSIFT) features and speeded-up robust features (SURF). However, oriented fast and rotated brief (ORB) features have outperformed both SIFT and SURF [151]. In the future, further analysis can investigate the ORB features on a breast cancer classification task.
- It will be important to investigate a reliable-performance CAD system over a longer period with various settings to understand the strengths and weaknesses to ensure the confidence and reliability of the system to be integrated in practical healthcare in the future of medical diagnosis.
- Recently, new algorithms like eXtreme Gradient Boosting (XGBoost) [153] have shown increased popularity because of their reliable performance and can be experimented with and integrated in CAD systems.
- Developing a mobile-based compatible expert system for breast cancer diagnosis to provide further convenience for more users to access, especially those with limited access to computer-based systems.

## Figures and Tables

**Table 2:** Comparison of adopted colour normalisation methods.

Ref	Year	Adapted Colour Normalisation Method
[42]	2017	Bejnordi et al. and Macenko et al. methodology
[31]	2017	Macenko et al. methodology
[27]	2018	Reinhard et al. and Kothari et al. methodology
[53]	2018	Macenko et al. methodology
[18]	2019	Macenko et al. methodology
[19]	2019	Macenko et al. methodology
[54]	2019	Reinhard et al. methodology
[29]	2019	Macenko et al. methodology
[55]	2019	Simplified version of Bejnordi et al. methodology
[22]	2021	Khan et al. methodology

**Table 3:** Comparison of data augmentation.

Ref	Flipping	Cropping/Shearing	Rotation	Translation	Shifting	Scaling	Zooming	Contrast	Fill Mode	Brightness
[11]	✓	✓	✓	✓	✓		✓			
[27]	✓		✓		✓					
[18]	✓	✓	✓	✓						
[53]		✓				✓				
[19]	✓	✓	✓				✓	✓	✓	
[17]	✓	✓	✓							
[21]	✓		✓		✓					✓
[26]	✓		✓							
[59]	✓	✓		✓		✓				
[60]			✓							
[20]	✓	✓	✓		✓				✓	

**Table 4:** Summary of segmentation approaches along with their definition, advantages, and limitations.

Approach	Segmentation Technique	Definition	Advantages	Limitations
Image processing	Region-based	Each pixel will be separated into groups (regions) in a homogeneous way based on a seed point.	Parameters are easy to be adjusted. Enables segmentation for multiple class. Beneficial for noisy images as their edges will be harder to detect.	Local region solution. Computationally expensive. Not robust enough. Missing/weak boundaries. Need to specify seed point. Output varies with different seed point.
	Edge-based	Edges are defined based on the sharp discontinuity (i.e., intensity) in the image.	Low computational complexity. Simple technique. Works fine with images with prominent edges.	Prone to over-segmentation error. Requires further morphological operation tuning for accurate result. Cannot apply on images with smooth edges. Requires high quality images. Hard to interpret with noisy images.
	Thresholding-based	Transform every pixel based on a threshold value obtained from a histogram of image that corresponds to regions.	Stable and flexible. Easy to implement.	Dependant on selection of an effective/correct threshold value. Not suitable for histology images because of the high complexity and various intensity distributions in images.
Machine learning	Cluster-based	Objects in image will be categorised into specific regions (groups) based on their similarity in pixels.	Efficient. Easy to implement. Solution dependant on the initialization. Able to preserve the information. Suitable for microscopic biopsy images.	Need to specify number of clusters. Sensitive to outliers.
	Energy-based optimization	Contour object of interest by minimizing/maximizing a predefined cos function.	High accuracy. Robustness.	High complexity. High computational time. Requires defining an effective cost function.
	Feature-based	Uses a model to train and learn the features to determine which pixels are ROI.	Supervised learning method. Robustness.	Application dependant.

**Table 5:** Summary of different segmentation approaches by several researchers.

Type of Technique(s) Employed	Ref	Year	Approach(es)	Remarks
Cluster-based	[82]	2013	Cluster algorithm	Contributed to a good feature extraction result.
	[83]	2015	K-means clustering algorithm	Able to preserve the desired information. Best suited for microscopic biopsy images.
	[84]	2017	Two step k-means clustering, and watershed transform	Performed on lymph nodes histology images. Considers local correlation of every pixel.
	[85]	2015	Segmentation: k-means clustering algorithm. Recover edges: Convex grouping algorithm.	Produces incorrect clusters when an image has fewer pixels of nuclei. Does not achieve splitting of overlapped cells.
Edge-based	[71]	2014	Watershed	Less complex and more computationally efficient. Prone to over-segmentation.
	[73]	2016	Distance regularized level set evolution (DRLSE) algorithm	Not sensitive to parameters. Able to detect and segment overlapping cells.
Energy-based optimization	[86]	2016	Graph cut: Spatio-colour-texture graph segmentation algorithm [95].	A novel integrated method. Similarity based super pixel generation method.
	[87]	2017	Hybrid active contour method	Accurate segmentation. Not sensitive to parameters.
	[88]	2017	Three-phase level set method to set contour	High accuracy in both clear and blurry nuclei images.
	[89]	2021	Watershed and improved gradient vector flow (GVF) snake model	Powerful segmentation model. Less prone to overlapping or obstructed boundaries
Feature-based	[94]	2015	Support vector machine (SVM)	Manually selects positively and negatively stained pixels from a set of representative images.
	[91]	2015	Multi-scale convolutional network	Fully automated segmentation process.
	[92]	2016	Distributed deep neural network	Fully automated segmentation process. High sensitivity of preserved images.
	[62]	2016	Cellular neural network (CNN) trained on genetic algorithm (GA) parameters	Prior information used to reduce errors.
	[93]	2019	Deep learning using HoVer-Net	Based on horizontal and vertical distance maps.
	[59]	2020	Faster R-CNN	A new method that has not been fully explored in breast cancer segmentation of mitosis cell. Computationally inexpensive. High accuracy.
Region-based	[62]	2015	Automated region growing using ANN to obtain threshold	Efficiently select threshold value to reduce errors.
	[65]	2015	Mean-shift algorithm	Application dependant. Able to handle arbitrary feature spaces.
	[63]	2016	Split and merging algorithm based on adaptive region growing	Requires a decent quality image. Fast and accurate when image is in good condition. Able to handle noise in image.
Threshold-based	[77]	2015	Maximally Stable Extreme Regions (MSER)	Accurate segmentation on complicated images.
	[78]	2017	Multilevel thresholding based on Grey Wolf Optimizer (GWO) algorithm using Kapur’s entropy and Otsu’s between class variance functions.	More stable and yields solutions. Performs faster than BFO, however slower than the PSO-based method.
	[76]	2015	Histogram-based thresholding	Hard to determine a suitable threshold.
	[79]	2015	Dictionary, thresholding	This is a mitotic cell detection system using a dictionary of cells.
	[75]	2017	Otsu thresholding	Does not require definition of many parameters.

**Table 6:** Comparison of different methods for breast cancer diagnosis system.

Approach on CAD Method	Ref	Year	Dataset	Classification Type	Methods	Results
Conventional	[83]	2015	2828 histology images	Binary	KNN	Accuracy: 92.2% Specificity: 94.02% Sensitivity: 82% F1-measure: 75.94%
	[126]	2015	Firat University Medicine Faculty Pathology Laboratory	Multi-class	SVM (Least Square Support Vector Machine)	Accuracy: 100%; four FN for benign tumours in a three-class problem
	[15]	2016	BreaKHis	Multi-class	SVM, Random Forest, QDA (Quadratic Discriminant Analysis), Nearest Neighbour	Accuracy: 80% to 85%
	[23]	2016	BreaKHis	Binary and Multi-class	SVM	Highest F1-score: 97.9%
	[128]	2016	Wisconsin Breast Cancer dataset	Binary	Decision tree: C4.5 algorithm	Accuracy: 91.13%
	[128]	2016	Wisconsin Breast Cancer dataset	Binary	SVM	Accuracy: 97.13%
	[30]	2019	BreaKHis	Binary	SVM	Prr: 92.1%
	[22]	2021	BreaKHis	Binary and Multi-class	Ensemble Classifier	Highest accuracy: 99%
Deep Learning	[26]	2018	BreaKHis	Binary and Multi-class	SVM and CNN	Accuracy: 96.15–98.33% (binary); 83.31–88.23% (multi-class)
	[17]	2016	BreaKHis	Binary and Multi-class	Single-task CNN (malignancy); Multi-task CNN (magnification level)	Prr: 83.13%. Prr: 80.10%
	[24]	2016	BreaKHis	Multi-class	AlexNet CNN	Prr: 90%
	[30]	2019	BreaKHis	Multi-class	MIL-CNN (Multiple Instance Learning-CNN)	Prr: 92.1%
	[53]	2018	BACH	Binary and Multi-class	ResNet-50, InceptionV3, VGG-16 and Gradient boosted trees	Accuracy: 87.2% (for binary) and 93.8% (for multi) AUC: 97.3% Sensitivity: 96.5 Specificity: 88.0%
	[60]	2018	BreaKHis	Binary	VGG16, VGG19, ResNet5 and Logistic regression	Accuracy: 92.60% AUC: 95.65% Precision: 95.95%
	[149]	2017	BreaKHis	Multi-class	Modified AlexNet and DeCAF (Deep Convolutional Activation Feature)	Accuracy: 81.5–86.3%F1-score: 86.7%-90.3%
	[31]	2017	Bioimaging Challenge 2015	Binary and Multi-class	CNN and SVM	Accuracy: 83.3%(binary); 77.8% (multi-class) Sensitivity: 95.6%.
	[25]	2017	BreaKHis	Multi-class	Custom CSDCNN (Class Structure-based Deep Convolutional Neural Network) based on GoogLeNet	Accuracy: 93.2%
	[27]	2018	BreaKHis	Binary and Multi-class	ResNet CNN	Accuracy: 98.77% (Binary) Prr: 96.25% (Multi class)
	[145]	2019	MITOS-ATYPIA-14, TUPAC-16	Binary	Modified faster-RCNN	Precision: 76% Recall: 72% F1 score: 73.6%
	[18]	2019	BreaKHisBioimaging Challenge 2015	Multi-class	Inception and ResNet CNN (IRRCNN) and Gradient boosting trees	Accuracy: 99.5% (binary); 96.4% (multi-class)
	[29]	2019	BreaKHis	Multi-class	Pre-trained CNN (AlexNet, ResNet-18 and ResNet-50) and SVM	Accuracy: 96.88% Sensitivity: 97.30% Specificity: 95.97% AUC: 0.9942
	[54]	2019	Bioimaging Challenge 2015	Multi-class	Pre-trained ResNet50 with SVM classifier	Accuracy: 95% Recall: 89%
	[28]	2019	BreaKHis	Binary	FCN (Fully Convolutional Network) based on AlexNet and Bi-LSTM (Bidirectional Long Short-Term Memory)	Accuracy: 91.90% Sensitivity: 96.8% Specificity: 91%
	[11]	2019	BreaKHis andBioimaging Challenge 2015	Binary and Multi-class	Inception Recurrent Residual CNN (IRRCNN)	Accuracy: 99.05% (for binary) and 98.59% (for multi)
	[55]	2019	Camelyon16	Binary	LYNA algorithm based on Inception-v3	AUC: 99% Sensitivity: 91%
	[19]	2020	BACH, BreaKHis, PatchCamelyon, and Bioimaging 2015	Binary	Pre-trained VGG19, MobileNet, and DenseNet with MLP (Multi-Layer Perceptron)	Accuracy: 92.71% Precision: 95.74% Recall: 89.80% F-score: 92.43%
	[21]	2020	BreaKHis	Multi-class	CNN features with MLP (Multi-Layer Perceptron)	Accuracy: 98.80%
	[59]	2020	MITOS-12MITOS-ATYPIA-14	Multi-class	Faster-RCNN and a score-level fusion of Resnet-50 and Densenet-201 CNNs	Precision: 87.6% Recall: 84.1% F1-measure: 85.8%
	[20]	2020	BreaKHis	Multi-class	BMIC_Net: Pre-trained AlexNet and KNN	Accuracy: 95.48%
	[22]	2021	BreaKHis	Binary and Multi-class	Xception and DenseNet CNNs	Accuracy: 99% (binary); 92% (multi-class)

## Data Availability

The data presented in this study are available in [15,31,32,33,35,36,37,38,39].
